# R-Ras regulates β_1_-integrin trafficking via effects on membrane ruffling and endocytosis

**DOI:** 10.1186/1471-2121-11-14

**Published:** 2010-02-18

**Authors:** Matthew W Conklin, Aude Ada-Nguema, Maddy Parsons, Kristin M Riching, Patricia J Keely

**Affiliations:** 1 Dept of Pharmacology, Laboratory for Molecular Biology and the University of Wisconsin Carbone Cancer Center, University of Wisconsin, 1525 Linden Dr, Madison, WI, 53706, USA; 2 Randall Division of Cell and Molecular Biophysics, King's College London Guy's Campus, London, SE1 1UL, UK

## Abstract

**Background:**

Integrin-mediated cell adhesion and spreading is dramatically enhanced by activation of the small GTPase, R-Ras. Moreover, R-Ras localizes to the leading edge of migrating cells, and regulates membrane protrusion. The exact mechanisms by which R-Ras regulates integrin function are not fully known. Nor is much known about the spatiotemporal relationship between these two molecules, an understanding of which may provide insight into R-Ras regulation of integrins.

**Results:**

GFP-R-Ras localized to the plasma membrane, most specifically in membrane ruffles, in Cos-7 cells. GFP-R-Ras was endocytosed from these ruffles, and trafficked via multiple pathways, one of which involved large, acidic vesicles that were positive for Rab11. Cells transfected with a dominant negative form of GFP-R-Ras did not form ruffles, had decreased cell spreading, and contained numerous, non-trafficking small vesicles. Conversely, cells transfected with the constitutively active form of GFP-R-Ras contained a greater number of ruffles and large vesicles compared to wild-type transfected cells. Ruffle formation was inhibited by knock-down of endogenous R-Ras with siRNA, suggesting that activated R-Ras is not just a component of, but also an architect of ruffle formation. Importantly, β_1_-integrin co-localized with endogenous R-Ras in ruffles and endocytosed vesicles. Expression of dominant negative R-Ras or knock down of R-Ras by siRNA prevented integrin accumulation into ruffles, impaired endocytosis of β_1_-integrin, and decreased β_1_-integrin-mediated adhesion. Knock-down of R-Ras also perturbed the dynamics of another membrane-localized protein, GFP-VSVG, suggesting a more global role for R-Ras on membrane dynamics. However, while R-Ras co-internalized with integrins, it did not traffic with VSVG, which instead moved laterally out of ruffles within the plane of the membrane, suggesting multiple levels of regulation of and by R-Ras.

**Conclusions:**

Our results suggest that integrin function involves integrin trafficking via a cycle of membrane protrusion, ruffling, and endocytosis regulated by R-Ras, providing a novel mechanism by which integrins are linked to R-Ras through control of membrane dynamics.

## Background

R-Ras is a small GTPase of the Ras family that plays a role in the transformation of various cell types [1-3], has been identified as a cancer-linked gene [4], and promotes tumorigenesis and metastasis of various carcinoma cells in vivo [5,6]. R-Ras is of particular interest to the study of integrin function, as it regulates cell adhesion through effects on integrin affinity and avidity, focal adhesion formation, and downstream signaling events [7-11]. This is likely bi-directional, as R-Ras is activated downstream of integrin-mediated adhesion as well [12]. R-Ras enhancement of cell adhesion is in opposition to the effects of H-, N-, and K-Ras, despite the fact that R-Ras has a near identical effector domain to these Ras molecules [1,3,13-15] and shares common effectors including Raf, PI_3_K, RalGDS, Nore-1, and PLCε [7,16-20]. To date, only one effector exclusive to R-Ras that is not shared with other Ras family members, RLIP76, has been identified [21]. Thus, the basis upon which R-Ras carries out its unique regulation of integrin function may lie in the subcellular localization of the molecule, for this likely defines the accessibility of R-Ras to defined subsets of upstream activators and downstream effectors. Currently, there has been little spatio-temporal characterization of R-Ras localization in living cells.

Ras family proteins are prenylated on their C-terminus "CAAX" motif by addition of a farnesyl or geranylgeranyl lipid group. This allows Ras proteins to target to membranes, and the precise configuration of additional motifs in the C-terminus, such as poly-basic domains or palmitylated cysteines, dictates the specific membrane subdomain localization of Ras family members [22]. Moreover, Ras proteins are internalized into the endosomal compartment, where their signaling capacity is sustained [23,24]. For H-Ras, this trafficking occurs through Rab 5- and Rab11- positive endosomes [25].

Rab GTPases are known to regulate endosomal compartment formation, function, and vesicular trafficking of proteins back and forth between the ER, golgi, PM, endosomes, and lysosomes [26]. The diversity of Rab subtypes helps to provide specificity to these many tasks, but there is overlap of function amongst Rab members [27-29]. Rab11 is found in recycling endosomes and has been observed to participate in the vesicular recycling of H-Ras, acetylcholine receptors, β-adrenergic receptors, integrins, and E-cadherin [25,30-33]. In particular, Caswell et al. [34] found that Rab25/Rab11c specifically directs recycling of α_5_β_1 _integrins from a vesicular pool located at the leading edge of migrating cells to the pseodopodial tips of cells.

Integrins are cell-surface adhesion receptors composed of an α and a β subunit, whose combination determines extracellular matrix ligand specificity. As with many other membrane receptors, integrins can be recycled through the cell and back to the plasma membrane, a process proposed to bring integrins from the rear of a migrating cell back up to the front [35]. Integrin recycling occurs through an endocytic pathway that involves Rab11 and Rab4 [32,36], and, in epithelial cells, Rab25 (also known as Rab11c) [34]. Importantly, the function of these Rabs are important for integrin-mediated adhesion, spreading, and invasion [32,34,36], suggesting an important role for integrin recycling in these processes. In addition, it is clear that association of integrins with a dynamic actin cytoskeleton and the ability to cluster laterally is important to integrin function [37,38]. Integrins have been noted to cluster into specific membrane subdomains, such as lipid rafts or caveolae [39], which is functionally important in integrin-mediated signaling and the endocytosis of integrins [40,41]. Based on these various observations, an attractive model is one in which integrins cluster into membrane domains, and are either engaged by the extracellular matrix resulting in a focal adhesion, or recycled if no ligand is locally available so that they can be used elsewhere on the cell surface. Other investigators have explored this idea where they found that integrins found on the dorsal surface of a cell migrating on a 2D substratum, which do not encounter ligand, are instead recycled into the cell [42], as are integrins that are clustered by antibody but not adherent to an ECM ligand [43]. The molecular regulation of these integrin trafficking patterns is not fully elucidated.

In addition to its effects on integrin function, R-Ras regulates membrane protrusion, cell migration, and cell spreading through effects on the actin cytoskeleton [10,12,16,18-20,44,45]. R-Ras is preferentially located to the leading edge of a migrating cell [12], where it controls the activation state of Rho, ROCK, and Rac in a coordinated fashion [12,46-48], suggesting that R-Ras might have an important role in regulating events at the plasma membrane. Thus, we set out to investigate whether R-Ras effects on membrane dynamics relate to its effects on cell adhesion through controlling integrin trafficking within the cell. We find that R-Ras and integrins co-localize to membrane ruffles and are internalized into Rab11 positive endosomes, and further that R-Ras activation is necessary for membrane ruffling, the endocytosis of integrins, and cell adhesion.

## Results

### Plasma membrane ruffling is dependent on endogenous R-Ras

**Figure 1:**
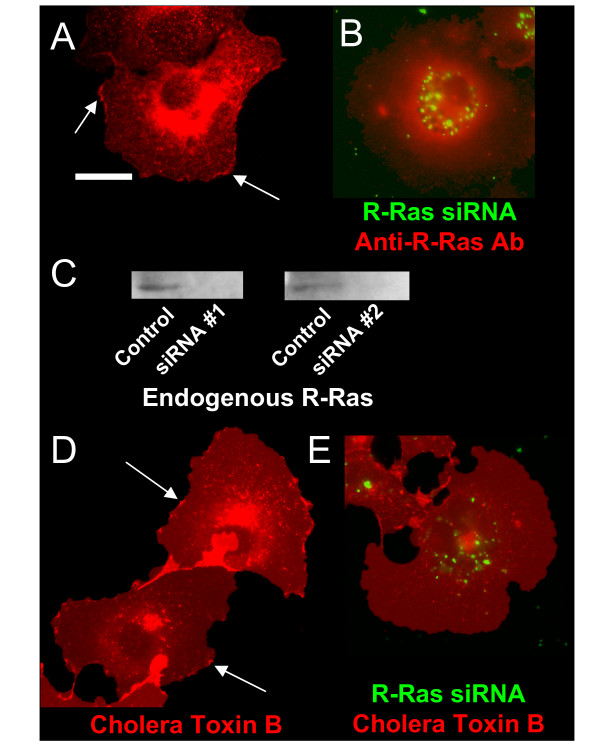
**Endogenous R-Ras and β_1_-integrin localize to membrane ruffles**. **(A) **The localization of endogenous R-Ras in Cos7 cells was determined by immunocytochemistry using antibody specific for R-Ras, which assembled into ruffles (arrows). **(B) **Immunofluorescence of a cell containing fluorescently labeled siRNA directed against R-Ras shows a loss of R-Ras from the plasma membrane, which occurred in 30/30 cells imaged. Perinuclear staining with this antibody in both A and B is non-specific, as determined by peptide competition experiments (not shown). **(C) **Western blot analysis to demonstrate R-Ras knock-down by transfection of either of two siRNA sequences targeting R-Ras (20 nM). **(D-E) **Imaging of fluorescent cholera toxin B-stained cells shows localization to ruffles. This localization was lost in 13/15 cells upon knockdown of R-Ras with siRNA.

Consistent with its role in cell protrusion and spreading, endogenous R-Ras was found at the membrane in ruffling microdomains (Fig. 1A, arrows) in immunofluorescently labeled Cos-7 cells. Ruffles were detected in 82% of cells stained in this manner (summarized in Table 1), where there were 2.9 ± 0.5 ruffles per cell on average. Expression of R-Ras could be knocked down using 20 nM siRNA directed against R-Ras (Fig. 1B-C), which caused a complete loss of ruffling in 30/30 cells imaged (Fig. 1B). Comparable results were obtained using a second independent R-Ras siRNA sequence. Using an independent, fluorescently tagged marker for membrane ruffling, cholera toxin B, which selectively binds to ganglioside-G_m1 _[49], concomitant results were obtained in 13/15 unfixed cells where ruffling was lost in cells with R-Ras knockdown (Fig. 1D vs. 1E and Table 1).

**Table 1:** Ruffle Summary Statistics

Stain	% of cells with ruffles	Ruffles per cell (+/- S.D.)
Endogenous R-Ras	42 of 51 **(82%)**	2.9 ± 0.5
with R-Ras siRNA	0 of 30 **(0%)**	N/A
Cholera Toxin B	29 of 34 **(85%)**	3.3 ± 0.7
with R-Ras siRNA	2 of 15 **(9%)**	0.3 ± 0.02
GFP-R-Ras(wt)	150 of 189 **(79%)**	3.2 ± 0.6
GFP-R-Ras(38V)	191 of 210 **(91%)**	8.9 ± 1.1
GFP-R-Ras(41A)	5 of 202 **(2%)**	0.1 ± 0.003

### R-Ras activation promotes ruffle formation and intracellular vesicle formation

**Figure 2:**
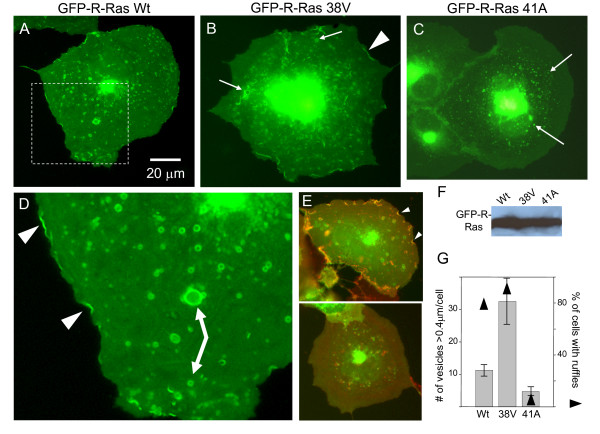
**GFP-R-Ras localizes to discrete plasma membrane domains**. Cos-7 cells were transfected with GFP-R-Ras constructs, and the localization of each to the plasma membrane determined. Plasma membrane localization was noted in 79% of GFP-R-Ras(wt) **(A**, n≥100 cells for each) and in 91% of constitutively active GFP-R-Ras(38V) **(B) **but not in cells transfected with dominant negative GFP-R-Ras(41A) **(C**, < 5% of cells). In all three transfectants, there was a variable amount of perinuclear fluorescence which included small, dot-like vesicles positive for GFP-R-Ras (arrows in C). In addition, both wt and 38V-expressing cells contained medium- and large-sized vesicles (≥0.4 μm). **(D) **3× zoomed view of dashed box in (A) highlights the appearance of plasma membrane fluorescence (arrowheads) and the assortment of vesicle and endosomal compartment sizes (arrows). **(E) **A cell transfected with GFP-R-Ras(38V) was co-stained with 4 μg/mL Alexa Fluor-555 cholera toxin B (top panel) to reveal co-localization of R-Ras with ganglioside-GM1 in ruffles (arrowheads). The lack of ruffle formation in GFP-R-Ras(41A) cells was independently confirmed by co-staining with cholera toxin B where ruffles were observed in 0 of the 26 cells imaged. **(F) **Western blot for GFP-R-Ras shows that protein levels were the same in GFP-R-Ras-(wt), -(38V) and -(41A) transfected cells in spite of the dramatic differences in localization of each isoform. **(G) **The number of endocytic compartments per cell was measured in >100 cells for each of the transfected constructs (bars). The percentage of cells that contained GFP-R-Ras localized to ruffles was quantified (triangles).

In order to assess the impact of R-Ras activation on ruffling dynamics, Cos-7 cells were transfected for 24 hrs with wild type (wt), constitutively active (38V), or dominant negative (41A) forms of GFP-linked R-Ras, and live cells were imaged over time by epifluorescence microscopy (Fig. 2). In all three transfectants, GFP-R-Ras localized to the perinuclear region, which was surrounded by numerous small (~0.35 μm) dots of R-Ras fluorescence. In addition, both GFP-R-Ras(wt) (Fig. 2A, E) and GFP-R-Ras(38V) (Fig. 2B) were localized in plasma membrane ruffles and in medium and large vesicular compartments within the cell. These two additional features are highlighted in the inset of the R-Ras(wt) image (Fig. 2E, arrowheads and arrows). In contrast, less than 5% of cells demonstrate localization of dominant negative GFP-R-Ras(41A) to membrane ruffles (Fig. 2C). As a control, cells transfected with GFP alone show no localization of fluorescence (Additional file 1, Figure S1). As in Figure 1D, we used a second marker for ruffles, cholera toxin B, so that we could determine whether dominant negative R-Ras regulates ruffles *per se*. Cells transfected with GFP-R-Ras(38V) and co-stained with 4 μL/mL Alexa555 cholera toxin B (Fig. 2E, top panel) demonstrated excellent registry between the two fluorophores at ruffles (arrowheads). However, cholera toxin B staining of cells transfected with GFP-R-Ras(41A) contained no ruffles (Fig. 2E, bottom panel), demonstrating that without proper R-Ras membrane targeting, ruffles do not form. All three cell types contained similar levels of GFP-R-Ras protein as assessed by western blotting (Fig. 2F) confirming that the different localization of R-Ras(wt) or R-Ras(38V) relative to R-Ras(41A) was not due to different levels of R-Ras expression.

The fluorescence at the plasma membrane was confined to ruffling microdomains that ranged in size between 0.5 and 12 μm and persisted for 30 seconds up to 20 minutes (see Additional files 2, 3, 4, Movies S1-3 and Additional file 5, Figure S2). Similar plasma membrane localization has been observed for other members of the Ras superfamily [24,50,51]. The percentage of cells with ruffles containing constitutively active GFP-R-Ras(38V) was greater than for cells expressing R-Ras(wt) (91% versus 79% respectively, n>100 cells for each, summarized in Table 1). Moreover, the number of GFP-R-Ras containing vesicles per cell was also increased in GFP-R-Ras(38V) cells (Fig. 2G).

In cells transfected with the dominant negative R-Ras(41A), in addition to the lack of fluorescence in ruffles, there was also a lack of medium and large sized R-Ras positive vesicles. These ring-shaped structures (arrows in Fig. 2E; see also Additional files 2, 3, 4, Movies S1-3), which ranged in size from 0.4 to 5 μm, are consistent with reports of trafficking vesicles and endosomal recycling compartments. Since they were absent in R-Ras(41A) cells, the medium and large compartments may be the result of the endocytosis of R-Ras from the plasma membrane. Consistent with this idea, the percentage of cells containing the largest (>1.2 μm) vesicles is higher in 38V cells than in wt (74% versus 88%, n>100 cells for each).

The trafficking of dynamic vesicles was dependent upon an intact actin cytoskeleton and myosin, as the addition of 30 mM 2,3-butanedione monoxime (BDM) to inhibit actin-myosin, or the use of the actin disrupting agents, 10 μM cytochalasin or 5 μM latrunculin A all arrested trafficking of GFP-R-Ras (see Additional file 6, Movie S4 for BDM).

### R-Ras endocytosis and trafficking occurs through Rab11- positive endosomal acidic compartments

**Figure 3:**
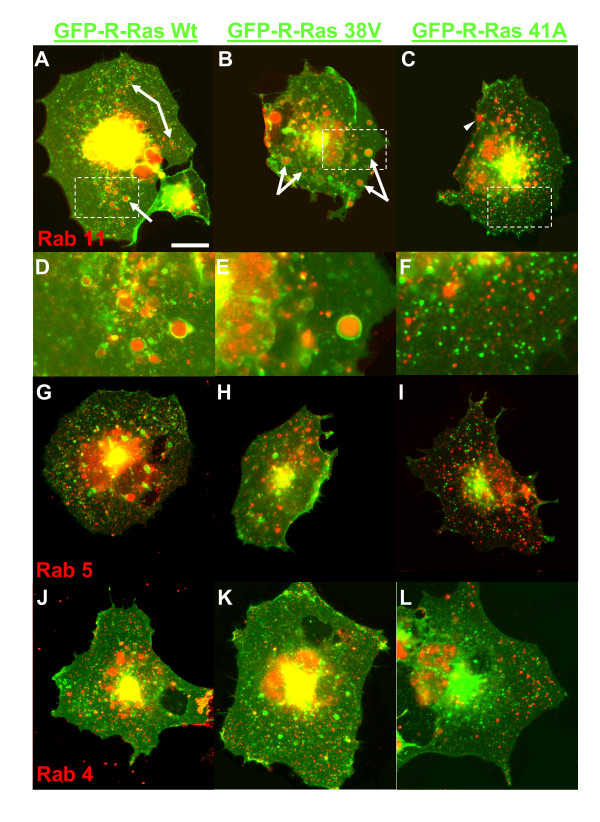
**GFP-R-Ras colocalizes with Rab11, but not Rab5- or Rab4-positive endosomal compartments**. Cos7 cells were transfected with GFP-R-Ras-(wt), -(38V), or -(41A) and then fixed and stained with antibodies against: **(A-F) **Rab11; **(G-I) **Rab5; or **(J-L) **Rab4. The largest vesicle compartments in 85% of -(wt) and 92% of -(38V) transfected cells were positive for Rab11 (arrows, for -(wt) n = 13 cells and -(38V), n = 12), but not Rab5 or Rab4 (<5%, n = 15 each). Intracellular GFP-R-Ras localized to smaller compartments and PM fluorescence lacked correlation with Rab fluorescence. Scale bar is 20 μm. **(D-F) **Insets of cells shown in A-C are shown at 3× zoom to highlight features.

We hypothesized that larger intracellular compartments in R-Ras(wt) and R-Ras(38V)-expressing cells are formed as a result of endocytosis and trafficking of R-Ras from ruffles via endosomal compartments. In GFP-R-Ras-expressing cells, many of the larger compartments were positive for Rab11 and are therefore likely to be endosomes (Fig. 3A and 3B, arrows). At least one trafficking compartment showed co-incidence of GFP-R-Ras and Rab11 in 85% of R-Ras(wt) and 92% of R-Ras(38V)-expressing cells. Smaller sized vesicles positive for GFP-R-Ras did not co-localize with Rab11. Interestingly, while the Rab11 vesicles were still present in cells expressing dominant negative R-Ras, Rab11 compartments were not associated with inactive GFP-R-Ras(41A) (Fig. 3C, arrowhead), supporting the notion that GTP-binding regulates R-Ras trafficking into this endosomal compartment, but that R-Ras does not itself regulate this compartment. These aspects are easier to observe in the higher magnification images of the boxed regions (Fig. 3D-F).

Rab5 is associated with clathrin mediated endocytosis from the PM to the early endosome, while Rab4 governs vesicular traffic at early endosomes [52]. However, neither Rab5 nor Rab4 showed correlation with GFP-R-Ras (Fig. 3G-L). Rab11-positive compartments did not account for all the GFP-R-Ras found in large (>1.2 μm) vesicles, and clearly there were many Rab5, 4, and 11 positive compartments unassociated with R-Ras. Our observation of R-Ras trafficking through endomembranes corroborates the recent findings of others where R-Ras was found to regulate the activation of RalA on endosomes [53].

The lumen of endosomes, lysosomes and vesicles is acidic and can be fluorescently labeled with Lysotracker Red DND-99 (Additional file 7, Figure S3A-C). Large GFP-R-Ras(wt) and (38V)-positive vesicles co-labeled with Lysotracker. Because of this co-labeling, as well as their large size and relative immobility, these vesicles are likely to be end-stage compartments such as lysosomes. Consistent with the lack of large vesicles in cells transfected with dominant negative R-Ras, GFP-R-Ras(41A) did not co-localize with Lysotracker.

Apart from the Rab5-regulated clathrin-coated pit pathway, another major endocytic pathway is via a caveolin-1 containing pathway. The invaginations attributed to caveolin-1 function are very small (~100 nm), [39,54]. We did not observe colocalization between R-Ras and caveolin-1 (Additional file 7, Figure S3D-F), suggesting that R-Ras does not traffic through a caveolin-1 positive compartment in these cells.

### Endocytosis of β_1_-integrin from the plasma membrane is governed by R-Ras

**Figure 4:**
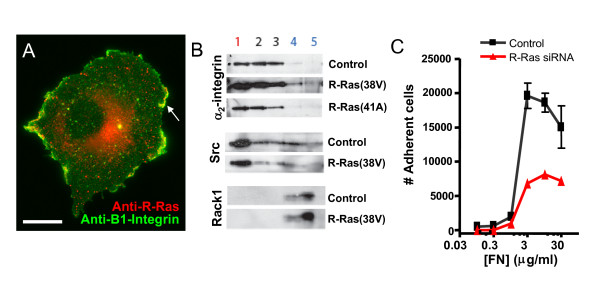
**R-Ras regulates integrin localization in the plasma membrane**. **(A) **Immunofluorescence of fixed Cos7 cells using antibodies against R-Ras and β_1_-integrin reveal colocalization of endogenous R-Ras and β_1_-integrin at ruffles. Scale bar = 20 μm. **(B) **R-Ras activation enhances integrin localization to low-density lipid fractions. Lysates from T47D cells stably expressing R-Ras(38V), R-Ras(41A) or control (vector only) were fractionated on an Optiprep™ gradient, and analyzed by subsequent SDS-PAGE and immunoblotting. Activated R-Ras increased the amount of β_1_-integrin (determined by antibody to the α_2 _subunit) at the plasma membrane, specifically in the low-density microdomain that contains Src, which itself was not increased in this membrane fraction by activation of R-Ras. Integrin was not found in the bulk plasma membrane, identified by Rack1. **(C) **Cos7 cell adhesion on increasing fibronectin (FN) concentrations was reduced following the transfection of 20 nM siRNA directed against R-Ras.

As integrin-mediated adhesion has been shown to be regulated by R-Ras [9], and both R-Ras and integrins traffic through a Rab11 positive compartment (Figure 3 for R-Ras and [32,36] for integrins), the relationship between integrins and R-Ras was examined more directly. Endogenous R-Ras and β_1_-integrin were co-localized in ruffles in 20/20 cells imaged (Fig. 4A, yellow). To assess the biochemical nature of this domain, and the effect of R-Ras activation on integrin localization to it, membranes were extracted and fractionated on a discontinuous gradient of iodoixanol (Optiprep™). Expression of constitutively active R-Ras(38V) increased the amount of α_2_β_1 _integrin at the plasma membrane, and in particular in the low-density fractions (Fig. 4B). These fractions are positive for Src, a myristoylated kinase known to traffic to lipid "rafts" or microdomains [55,56], and are distinct from those positive for Rack1, a marker of bulk plasma membrane [41]. This regulation of integrin localization to lipid domains is likely functionally relevant, as cells transfected with siRNA directed against R-Ras had significantly reduced cell adhesion (Fig. 4C), consistent with previous results [12,16].

**Figure 5:**
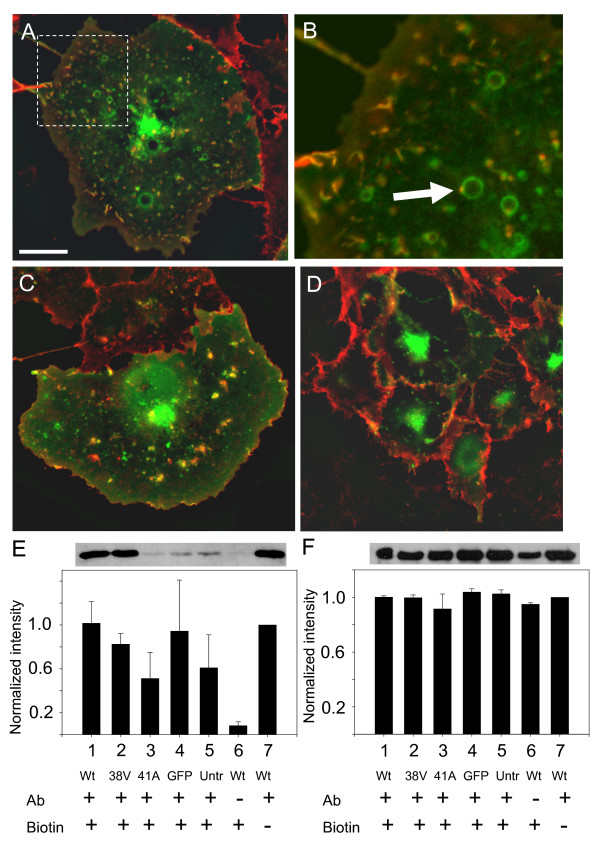
**β_1_-integrin is endocytosed from ruffles in an R-Ras dependent manner**. Alexa Fluor555 (red)-labeled β_1_-integrin antibody was applied to live Cos7 cells expressing: **(A-B) **GFP-R-Ras-(wt), **(C) **-(38V), or **(D) **-(41A). There was significant overlap between β_1_-integrin and R-Ras (yellow) at ruffles and in vesicles in GFP-R-Ras(wt) and GFP-R-Ras(38V) cells, which was absent in GFP-R-Ras(41A) cells. Note the uniform distribution of β_1_-integrin in cells expressing GFP-R-Ras(41A) **(D)**. Scale bar is 20 μm. **(E-F) **Cells were transfected with the following constructs; lanes 1, 6, and 7- GFP-R-Ras(wt), lane 2- GFP-R-Ras(38V); lane 3- GFP-R-Ras(41A), lane 4- GFP, lane 5- untransfected. Following a 30 min incubation with β_1_-integrin antibody (with the exception of lane 6 as a control, where cells were exposed to antibody for <1 min), cells were biotinylated (with the exception of lane 7, as a control), washed, then lysed and cleared of the biotinylated antibody using strep-avidin beads. The presence of any remaining antibody would be a result of endocytosis during the incubation period, and was quantified by western blot. Densitometry of bands was normalized to lane 7 (wt with no biotinylation) and averaged data (± SEM) from three separate experiments is shown in E. Panel F is data from two experiments where whole cell lysate was probed for β_1_-integrin as a loading control. Exemplar blots are shown above the bar graphs.

We next investigated the dynamics of integrin and R-Ras trafficking by live cell imaging. Antibody against the β_1_-integrin was directly labeled with Alexa Fluor 555 so that localization of β_1_-integrin could be imaged without fixation and secondary antibody application. In GFP-R-Ras(wt) and (38V)-expressing cells, significant colocalization of β_1_-integrin and R-Ras in ruffles was observed (Fig. 5A-C). This co-localization was not noted in cells expressing GFP-R-Ras (41A) (Fig. 5D). Interestingly, the β_1_-integrin fluorescence in R-Ras(41A)-expressing cells was uniform around the entire cell periphery, but was conspicuously not localized within ruffles, in contrast to GFP-R-Ras(wt) or GFP-R-Ras(38V)-expressing cells. This was not due to changes in integrin levels, as the total, cell-wide expression level of integrins was similar (Fig. 5F). Therefore, expression of dominant negative R-Ras(41A) does not inhibit initial targeting of β_1_-integrin out to the plasma membrane, but rather impairs the clustering and subsequent endocytosis of β_1_-integrin, resulting in a net increase in cell surface β_1_-integrin. Thus, it is our hypothesis that integrins gather into and are endocytosed preferentially from membrane ruffles, whose formation is governed by activation and localization of R-Ras.

As an independent means of assessing the regulation of β_1_-integrin endocytosis via R-Ras, a cell-surface biotinylation-protection assay was used. Cells transfected with GFP-R-Ras constructs were exposed to β_1_-integrin antibodies for 30 mins followed by incubation of live cells with NHS-LC-biotin. Anti-β_1_-integrin antibody that was endocytosed during that 30 min should be protected from biotinylation. Following washout of unbound biotin, cells were lysed and the biotinylated antibody cleared using strep-avidin beads, leaving only the β_1_-integrin bound antibody that was endocytosed during the incubation period. Endocytosed β_1_-integrin antibody was quantified by western blotting and compared to total β_1_-integrin expression from whole cell lysates (Fig. 5E-F, densitometry of bands was normalized to the wt cells not treated with biotin, lane 7). GFP-R-Ras(wt) and GFP-R-Ras(38V)-expressing cells had comparable amounts of β_1_-integrin uptake (lanes 1 and 2, Fig. 5E), which was severely reduced in GFP-R-Ras(41A) transfected cells (lane 3). This result verifies the imaging data and further supports the hypothesis that β_1_-integrin endocytosis is regulated by R-Ras.

**Figure 6:**
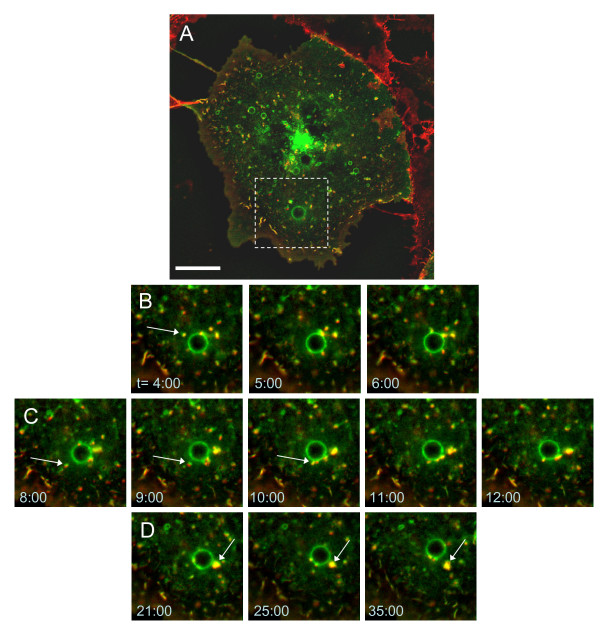
**β_1_-integrin traffics through R-Ras-positive vesicles**. Time course images of **(A) **a GFP-R-Ras(wt) transfected Cos7 cell incubated in Alexa Fluor555 (red) labeled β_1_-integrin antibody solution for 30 mins reveal the interplay of R-Ras and β_1_-integrin (See Additional file 8, Movie S5). **(B, C) **The inset of images from the boxed region in A acquired at one minute intervals show that R-Ras and β_1_-integrin traffic together to large endosomal vesicles. **(D) **Endocytosed components are then repackaged together and bud off from these organelles. Scale bar is 20 μm.

Observed over time, β_1_-integrin was endocytosed from ruffles together with GFP-R-Ras into vesicles that trafficked to the larger, endosomal recycling vesicles (arrow in 5B). This is illustrated in greater detail in Fig. 6 and Additional files 8, 9, 10, Movies S5-7, where a different region of interest (than the box in Fig. 5A) was examined over a 40 min time course. Images acquired at one minute intervals capture β_1_-integrin being endocytosed via GFP-R-Ras positive vesicles (Fig. 6B and 6C). Interestingly, there was not a total integration of β_1_-integrin into the entire vesicle, but rather the labeled integrin remained in discrete patches. Endocytosed R-Ras and integrin components were repackaged together and budded off the larger endosomal compartment, but on a longer time scale (Fig. 6D). The trafficking was intermittent, and not all large vesicles were observed to interact with β_1_-integrin or GFP-R-Ras trafficking vesicles during the imaging period. The observation of β_1_-integrin endocytosis via trafficking vesicles to endosomes has also been observed in MDA-MB-231 cells, where trafficking was dependent on the nucleotide binding state of Rab21 [57].

**Figure 7:**
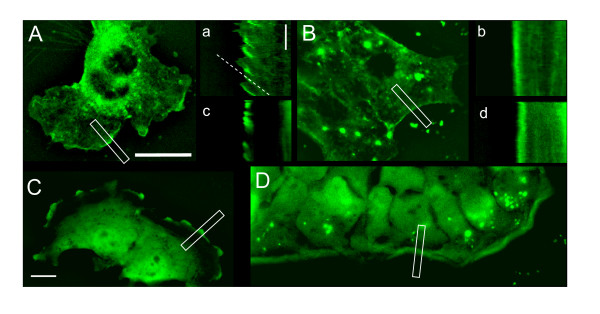
**Plasma membrane ruffling is dependent on R-Ras**. NMuMG cells stably transfected with GFP-β_1_-integrin **(A, a) **contained many ruffles (in 23/23 cells imaged) whose retraction from the cell edge can be observed in the accompanying kymograph (lowercase letters, corresponding to each panel). Cells transiently transfected with two different siRNA sequences targeting R-Ras did not have ruffles in 16/16 cells imaged **(B, b) **even though β_1_-integrin could be seen at thecell edge in portions of many cells. However kymography analysis of these areas reveals that the β_1_-integrin is dynamic in cells replete with endogenous R-Ras. **(C, c and D, d) **GFP-VSVG localizes to the bulk plasma membrane, suggesting that R-Ras has a more general effect on membrane ruffling. Ruffling that was observed in 15/15 cells imaged was abolished in 15/15 cells that were transfected with R-Ras siRNA. Scale bars = 20 μm (A, B, C, D) or 10 mins (a, b, c, d).

In order to confirm these results in a somewhat different system, and to more directly observe the dynamics of β_1_-integrin at the membrane, NMuMG cells were stably transfected with GFP-β_1_-integrin and timelapse images were acquired. GFP-β_1_-integrin was localized to prominent ruffles (Fig. 7A, representative of 23 different cells imaged). Accompanying kymography analysis (corresponding lowercase letters) demonstrated that several prominent protrusions of the cell edge occurred during a 30 min imaging period (Fig. 7a). Additionally, the bright fluorescence, indicating GFP-β_1_-integrin localization within ruffles, was spatially retracted towards the interior of cells over time. The slope of this line of fluorescence is a measure of the ruffle retraction speed, which was 1.47 ± 0.12 μm/min (S.E. n = 23). These results contrast those found in R-Ras siRNA transfected cells (Fig. 7B) where no ruffles were observed in 16 cells imaged for 30 mins each, and the kymograph shows that the plasma membrane of the cells was still, and that GFP-β_1_-integrin localization was unaltered over time (Fig. 7b). This result was an independent confirmation of the observations made in Fig. 5D and Additional file 10, Movie S7, where dominant negative R-Ras halted membrane dynamics and β_1_-integrin trafficking. In control cells, the fluorescence from GFP-β_1_-integrin in ruffles was observed to retract 2-8 μm from the cell edge before being disassembled into constituent vesicles. This is shown in Additional files 11 and 12, Movies S8 and S9, where ribbon-shaped retracted ruffles disintegrated into numerous smaller vesicles that exhibited movement directed towards the interior of the cell, presumably for recycling purposes. No such trafficking vesicles were observed in cells transfected with R-Ras siRNA.

To determine if R-Ras affects the dynamics of a protein localized to the plasma membrane in a manner similar to that of GFP-β_1_-integrin, NMuMG cells stably expressing GFP-VSVG (Fig. 7C) were imaged. While GFP-VSVG did localize to membrane ruffles (in all 15 live cells imaged), the image appearance was slightly altered from the GFP-β_1_-integrin expressing cells, in that the ruffles appeared to be thicker, which may reflect a greater expression and localization of GFP-VSVG to the plasma membrane. Moreover, kymography analysis (Fig. 7c) and Additional file 13, Movie S10 show the spontaneous appearance of GFP-VSVG in ruffles. However, unlike GFP-β_1_-integrin, the ruffle was not observed to retract into vesicles. Instead, GFP-VSVG moved laterally out of the ruffle and none of the endocytic vesicles that were noted for GFP-β_1_-integrin were observed for GFP-VSVG. Nevertheless, like GFP-β_1_-integrin, the ruffling dynamics of this membrane marker was also R-Ras dependent, as two different siRNA sequences directed against R-Ras each abolished ruffling and GFP-VSVG dynamics in all 16 cells imaged (Fig. 7D and 7d). We performed immunocytochemistry to examine the spatial relationship between VSVG, R-Ras, and β_1_-integrin and found that they are all colocalized in ruffles (Additional file 14, Figure S4, yellow, n>10 cells for each condition). These results suggest that R-Ras acts a general regulator of protrusion and membrane ruffling, but that the molecules within these ruffling domains such as β_1_-integrin or GPI-anchored proteins can have different subsequent recycling fates.

## Discussion

We describe here a role for R-Ras in the regulation of integrin trafficking and membrane dynamics. Endogenous R-Ras and β_1_-integrin co-localize to ruffles that are lost when R-Ras is knocked down by siRNA. R-Ras activation is necessary for the formation of ruffles, since their formation is lost in cells expressing dominant negative R-Ras or siRNA directed to R-Ras, while their creation is enhanced in cells expressing constitutively active R-Ras. Following endocytosis of ruffles, both R-Ras and β_1_-integrin traffic together through a Rab11-positive endosomal compartment. The functional outcome of R-Ras activation is enhanced formation of a ruffling membrane, localization of integrins to that ruffle, and endocytosis of integrins. We propose that these steps are part of the mechanism by which R-Ras enhances cell adhesion, since loss of R-Ras function does not cause integrins to be lost from the cell surface, but rather prevents their dynamic clustering and recycling. Consistent with this notion, others have provided strong evidence that recycling *per se *is important for integrin function [58,59]. Here, we add the novel finding that R-Ras is a regulator of this event.

It is our hypothesis that the cycle of R-Ras dependent protrusion, ruffling and endocytosis serves as a sensor of the local microenvironment that allows cells to optimize functional adhesions. There are two possible outcomes for a clustered integrin at the cell surface, both of which are regulated by R-Ras: either it binds to ECM, or it fails to form a functional adhesion. In the case of integrin binding to a ligand, we envision that R-Ras-dependent protrusion facilitates the initial interaction between β_1_-integrin and the ECM, and also strengthens the adhesion by turning on Rho and ROCK [12]. Consistent with this, it has been shown that R-Ras promotes cell adhesion and spreading, in part through increases in integrin avidity [8,10,11,16]. Conversely, we propose that integrins that do not bind to ECM will be endocytosed through R-Ras-dependent plasma membrane ruffling, and subsequently transferred to Rab11 endosomal compartments through which integrins have previously been shown to traffic [32,34,36]. Endocytosis of unbound integrins could allow the rapid recycling of integrins back to the plasma membrane where they would regain the opportunity to participate at active sites of cell adhesion [60]. By increasing the rate at which this cycling occurs, active R-Ras may promote both possible outcomes: ruffling and focal adhesion formation. Consistent with this idea, integrins located on the dorsal surface of a cell migrating on a 2D substratum that do not encounter ligand are instead recycled into the cell [42], as are integrins that are clustered by antibody but not adherent to an ECM ligand [43]. Integrins that form functional focal adhesions are endocytosed by a clathrin mediated process dependent on dynamin and focal adhesion kinase [61,62]. In cells that express dominant negative R-Ras, β_1_-integrin remains on the surface and does not cluster, and moreover is unprotected from surface biotinylation (Figure 5). Furthermore, GFP-β_1_-integrin is immotile in cells transfected with R-Ras siRNA, underscoring the necessity of R-Ras for β_1_-integrin dynamics. Thus, by facilitating integrin endocytosis and membrane dynamics, R-Ras may advance the spatial localization of integrins to relevant functional sites, thereby promoting cell adhesion.

We have previously observed that R-Ras regulates actin-based lamellipodial protrusions [12,16]. It is therefore worth noting that endosomal trafficking is also influenced by actin dynamics [63,64]. The role of R-Ras in spatially regulating integrin activation may contribute to the finding that actin polymerization produces high-avidity integrins at the leading edge of migrating cells [37], which is the precise localization of R-Ras [12]. It is also likely that this regulation is bidirectional, as integrins regulate the targeting and internalization of membrane domains [41]. In this view, we propose that the effects of R-Ras on actin structure, membrane dynamics, and ruffling are likely coordinately related to one another in order to spatially enhance integrin clustering and thus avidity, resulting in the regulation of cell adhesion and migration.

By colocalizing with integrins at the plasma membrane, R-Ras may also facilitate the recruitment of signaling molecules proximal to integrins, which explains the observation that integrin-mediated signaling events are enhanced by R-Ras [10]. Indeed, R-Ras C-terminal membrane targeting motifs are required for integrin function [8]. Integrins within the protruding leading edge of cells could then coordinate the activity of co-assembled kinases and GTPases as needed. Because R-Ras has no enzymatic or kinase activity itself, functions ascribed to activated R-Ras are carried out by its effectors such as PI3K, PLCε, Raf or Ral [1,3,7,14-16]. We have found that both PLCε and PI3K, both regulators of phospholipids, appear to be important effectors for R-Ras effects on membrane ruffling, migration and cell spreading [12,16]. Thus, we propose that the downstream signaling from R-Ras is processed by the cell to coordinate motility, membrane protrusion, and cellular behavior in part through effects on integrin dynamics.

## Conclusions

We find that R-Ras regulates membrane ruffling, the localization and clustering of integrins into specific membrane subdomains, and the endocytosis of integrins. R-Ras and β_1_-integrin localize together at the plasma membrane and traffic together through a Rab 11 positive recycling compartment. Loss of R-Ras function results in β_1_-integrin that remains on the plasma membrane and fails to cluster or be endocytosed. The effect of R-Ras on integrin trafficking corresponds to the enhancement of integrin function by R-Ras, and represents an additional means by which R-Ras regulates integrins.

## Methods

### Cell culture and transfection

Cos-7 cells were obtained as a generous gift from Dr. Richard Anderson (Madison, WI) and maintained in DMEM (containing high glucose, L-glutamine, sodium pyruvate and pyridoxine hydrochloride) plus 10% fetal bovine serum at 5% CO_2_. T47D human breast carcinoma cells were obtained from ATCC, and NMuMG cells were obtained from Dr. Caroline Alexander (Madison, WI) and maintained as previously described [9,65]. Cells were plated into either uncoated 6-well plates or poly-L-lysine coated glass bottomed dishes (MatTek Ashland, MA) for 24 hours at low confluency to minimize cell-cell interactions. The full coding sequences of R-Ras human cDNAs were amplified by PCR and subcloned in frame into Bam HI/Xba I site of the mammalian expression vector pEGFP-C1 (Clontech, Mountain View, CA). Cells were transiently transfected with plasmid DNA encoding the full length wild type (Wt) R-Ras or, alternatively, cells were transfected with the GFP-tagged constitutively active form of R-Ras (38V) in which amino acid 38 was mutated from a glycine to a valine to prevent GAP (GTPase activating protein) from binding, or with GFP-tagged dominant negative R-Ras (41A) in which amino acid 41 was mutated from a glycine to an alanine to favor the GDP-binding state [3,13]. NMuMG (Normal Murine Mammary Gland) cells were initially transfected with GFP-β_1_-integrin (a gift from Maddy Parsons) or GFP-VSVG (Vesicular Stomatitus Virus G; Addgene; plasmid 11912) using Lipofectamine 2000, for stable expression cells were then selected using G418 followed by flow cytometry. Fluorescently tagged siRNA (Qiagen) for R-Ras was transfected into cells using Lipofectamine 2000 reagent for 24 hrs. Two different siRNA sequences were used with complimentary results (Target sequence #1 CCG GAA ATA CCA GGA ACA AGA; #2 CCG GGT CAC TGC TGT ATA TAA). Direct labeling of β_1_-integrin antibody (clone 4B7R, sc-9970, Santa Cruz) was carried out using a Zenon Alexa Fluor-555 mouse IgG_1 _labeling kit from Molecular Probes following the manufacturers protocol. Labeled antibodies were applied to cells for 30 mins; the excess was then washed out, followed by cell imaging.

### Epifluorescence imaging

Images were acquired using either a DP-70 digital camera (Olympus, Melville, NY) mounted to an upright microscope or a CoolSnap FX (Roper) CCD camera mounted to an inverted microscope working in the epi- mode. The emission intensity of a 100 W mercury arc bulb was attenuated by the appropriate excitation filter (480/30 nm for GFP, 560/55 for red fluorescence), directed to the cells with a dichroic mirror (505 DCLP for GFP, 595 DCLP for red), and fluorescence emission was filtered (535/40 for GFP, 645/75 for red) before reaching the camera. Exposure times ranged between 100-500 ms and a 20× N.A. = 0.95 water immersion, or 40× N.A. = 1.3 oil immersion objective was used. Digitized images were acquired using Slidebook (Olympus) imaging software and, when indicated, deconvolved using the No neighbors algorithm. Images were then exported to Image J for further analysis. Alexa Fluor-555 Cholera toxin B, and Lysotracker Red DND-99 were all acquired from Molecular Probes (Eugene, OR). Kymography analysis was performed using a custom plug-in installed in Image J. A 5 pixel wide line was drawn across the cell where the average intensity across the 5 pixels was compressed into a single line. This was done for each of 60 images acquired 30 seconds apart and the lines were stacked next to each other to create a distance versus time plot.

NOTE: It is recommended that the reader examine images in the digital document at greater than 100% zoom in order to best visualize the fine detail of subcellular localization described in this paper.

### Immunocytochemistry

The following protocol was utilized for all primary antibody targets studied: Cos-7 cells transfected with GFP-R-Ras constructs were rinsed in PBS to remove DMEM, fixed in 4% paraformaldehyde for 15 mins at room temperature (RT), followed by quenching of excess PFA in 0.15 M glycine for 10 mins. Cells were blocked in 1% donkey serum and 1% fatty acid-free BSA for 30 mins after which primary antibody was applied overnight at 4°C. All antibody solutions consisted of blocking solution supplemented with 0.01% Triton-X-100. Following washout of the primary antibody solution with PBS, the secondary antibody was applied for 1 hr at RT followed by several rinses with PBS. Cells were imaged the same day as secondary antibody application on the epifluorescence microscope described above.

The following primary antibodies were used: mouse anti-Rab11 at 1:100; mouse anti-Rab5 at 1:100; mouse anti-Rab4 at 1:100 (all from BD Biosciences Pharmingen); rabbit anti-caveolin-1 [Tyr-14 phospho-specific] (Cell Signaling Technologies, 1:100); human anti-β_1_-integrin (clone N29, Chemicon, 1:100; which recognizes an activation-dependent epitope and is described in Wilkins et al. [66]); human anti-R-Ras, custom made by Antibodies by Design (see below), 30 μg/mL. As the case dictated, either Alexa-Fluor-568 goat anti-mouse, anti-human, or Alexa-Fluor-546 goat anti-rabbit secondary antibodies from Molecular Probes were applied at 1:100 concentration.

### R-Ras antibody production

Bivalent C-terminally myc- and his-tagged mini-antibodies (Fab_dHLX-MH) were produced by Antibodies by Design (AbyD; Munich, Germany) by a proprietary process: antibodies were obtained by panning AbyD's HuCAL^® ^GOLD library against GDP- and GTPγS-loaded GST-R-Ras antigens that we purified and supplied. Antibodies were enriched by preadsorption against GST, then alternate panning (first and third round of selection on GDP-R-Ras, second round on GTPγS-R-Ras). Binding affinities of the resultant antibodies was profiled by enzyme-linked immunosorbent assay (ELISA). We identified 5 of 8 antibodies on the AbyD ELISA that had strong binding to R-Ras. These antibodies were further prepared and purified for use.

### Fractionation of cell membranes

T47D cells were lysed in TNE buffer (50 mM Tris-HCl, pH 7.4, 150 mM NaCl, 5 mM EDTA, 0.25% Triton-X-100) supplemented with protease inhibitor cocktail (Sigma, St. Louis) and 5 mM DTT. Lysates were extracted on ice for 20 min, and adjusted to 50% Optiprep™ (iodoixanol) before being overlaid with a 30% and a 0% Optiprep™ solution (prepared inTNE with 0.1% Triton-X-100). Samples were centrifuged at 40,000 rpm in an SW 65K rotor (Beckman) at 4°C. One mL fractions were collected from the top and analyzed by SDS/PAGE and western blotting.

### Biotinylation

Cos7 cells transfected and incubated with labeled β_1_-integrin antibody as described above were subsequently biotinylated (Pierce EZ-Link Sulfo-NHS-LC-Biotin, >20-fold molar excess) for 30 mins on ice. Following washout of unbound biotin, cells were lysed and biotinylated antibody was cleared using a 40 min incubation of strep-avidin beads and mild centrifugation for 20 seconds. Supernatant was incubated in gamma-bind sepharose beads (GE Healthcare BioSciences) for 40 mins followed by 3 rounds of mild centrifugation and resuspension. A final denaturation was performed using 1.5× Laemmli buffer containing β-mercaptoethanol at 100 degrees for 3 mins. Samples were run on an 8% SDS-PAGE gel, transferred to a PVDF membrane, probed for β_1_-integrin, and the 50 kDa band of the denatured antibody was quantified using Image J analysis software.

### Western blotting

Lysates were prepared from untransfected cells, or from cells transfected for 24 hours with the GFP-R-Ras constructs. Samples were run on a SDS-PAGE gel and transferred to a PVDF membrane which was probed with rabbit anti-R-Ras antibody (1:400, Santa Cruz) followed by anti-rabbit HRP (Jackson Immunoresearch laboratories, West Grove, PA 1:5000) and ECL substrate (Amersham Biosciences) followed by exposure to film.

### Cell adhesion assay

Calcein-AM loaded (300 nM) Cos7 cells were seeded into the wells of a 96-well plate (Greiner Bio One) that had been coated overnight with the indicated concentration of fibronectin as described (Keely et al., 1999). Cells that adhered after a 30 min incubation followed by washing were measured by a Tecan GENias Pro plate reader. Cells were either untransfected, or subjected to 20 nM R-Ras siRNA for 24 hours before plating. All measurements were done in triplicate, and results were plotted using linear regression analysis of standard curve data and plotted on a semi-log scale.

## Abbreviations

Abbreviations used in this paper: ECM: extracellular matrix; GDP: guanosine diphosphate; GFP: enhanced green fluorescent protein; GTP: guanosine triphosphate; NMuMG: normal mouse mammary gland; PBS: phosphate buffered saline; PM: plasma membrane; Wt: wild type.

## Authors' contributions

AA-N created the GFP-R-Ras constructs, and performed membrane fractionation experiments and pilot trafficking experiments; KMR created the NMuMg cells that stably express GFP-β_1_-integrin; MP created the GFP-β_1_-integrin construct; PJK conceived of the study, edited the manuscript, and is the principal investigator; MWC carried out all of the remaining experiments and drafted the manuscript. All authors read and approved the final version of this manuscript.

## Supplementary Material

Additional file 1 **GFP-alone control**. Representative cell that was transfected with GFP alone showed no localization of the fluorophore to membranes. Click here for file

Additional file 2 **Movie of GFP-R-Ras(wt) dynamics**. Timelapse images of GFP-R-Ras(wt) localization. Images were acquired at 2 min intervals for 20 mins and played at 7 frames/sec. Click here for file

Additional file 3 **Movie of GFP-R-Ras(38V) dynamics**. Timelapse images of GFP-R-Ras(38V) localization. Images were acquired at 2 min intervals for 20 mins and played at 7 frames/sec. Click here for file

Additional file 4 **Movie of GFP-R-Ras(41A) dynamics**. Timelapse images of GFP-R-Ras(41A) localization. Images were acquired at 2 min intervals for 20 mins and played at 7 frames/sec. Click here for file

Additional file 5 **GFP-R-Ras ruffle endocytosis**. Montage of the GFP-R-Ras(wt) cell in Figure 2A. Two separate ruffles can be seen to disappear over time coincident with the appearance of vesicle-like structures at the site of the former ruffle. Images were acquired at 2 minute intervals for 30 minutes and shown at 3× magnification compared to the original. Click here for file

Additional file 6 **Actin dependence of intracellular vesicle traffic**. Timelapse images of a GFP-R-Ras(41A) cell where the application of 30 mM 2,3-butanedione monoxime (BDM) to cells half way through the movie arrests dynamic intracellular movement. Images were acquired at 1 min intervals for 26 mins and played at 3 frames/sec. Click here for file

Additional file 7 **GFP-R-Ras is endocytosed to lysosomes via a non-caveolin-1 mediated pathway**. (A-C) Shown are enlarged images of unfixed cells transfected with GFP-R-Ras constructs that were incubated with 1 μM Lysotracker Red DND-99 for 20 mins followed by washout. All Lysotracker-positive compartments larger than 1.2 μm contained R-Ras (n = 7 GFP-R-Ras(wt) cells, n = 8 GFP-R-Ras(38V) cells). There were numerous, smaller compartments in all cell types with no correlation between GFP-R-Ras and Lysotracker. (D-F) Transfected cells were fixed and stained for phospho-specific (Tyr-14) caveolin-1, but the lack of correspondence with GFP-R-Ras suggests that GFP-R-Ras is endocytosed separate from caveolin-1. Scale bar is 20 μm. Click here for file

Additional file 8 **Movie of R-Ras and β_1_-integrin vesicle trafficking-Inset**. Timelapse images of GFP-Ras (green) and Alexa Fluor555 (red) labeled β_1_-integrin antibody. The movie is an inset of a GFP-R-Ras(wt)-expressing cell. Images were acquired at one minute intervals for 40 minutes and played at 7 frames/sec. Data was deconvolved using the no-neighbors algorithm. Click here for file

Additional file 9 **Movie of R-Ras and β_1_-integrin vesicle trafficking-Full view**. Timelapse images of GFP-Ras (green) and Alexa Fluor555 (red) labeled β_1_-integrin antibody. The movie is of a GFP-R-Ras(wt)-expressing cell. Images were acquired at one minute intervals for 40 minutes and played at 7 frames/sec. Data was deconvolved using the no-neighbors algorithm. Click here for file

Additional file 10 **Movie of GFP-R-Ras(41A) and β_1_-integrin vesicle trafficking**. Timelapse images of GFP-Ras (green) and Alexa Fluor555 (red) labeled β_1_-integrin antibody. The movie is of a GFP-R-Ras(41A)-expressing cell. Images were acquired at one minute intervals for 40 minutes and played at 7 frames/sec. Data was deconvolved using the no-neighbors algorithm. Click here for file

Additional file 11 **Movie of GFP-β_1_-integrin in membrane ruffles**. Movie of GFP-β_1_-integrin transfected cells illustrate ruffling and endocytosis. Images were acquired 30 seconds apart for 30 mins. Data was deconvolved using the no-neighbors algorithm. Click here for file

Additional file 12 **Endocytosis of β_1_-integrin through the breakdown of ruffles**. Inset view of movie 7 showing GFP-β_1_-integrin fluorescence breakdown into vesicles following ruffling. Images were acquired 30 seconds apart for 30 mins. Data was deconvolved using the no-neighbors algorithm. Click here for file

Additional file 13 **GFP-VSVG**. Movie of a GFP-VSVG transfected cell with a ruffling membrane. Images were acquired 30 seconds apart for 30 mins. Data was deconvolved using the no-neighbors algorithm. Click here for file

Additional file 14 **R-Ras, β_1_-integrin, and VSVG colocalize within ruffles**. Cells were transfected either with GFP-VSVG or GFP-β_1_-integrin, and then stained with anti-R-Ras or anti-β_1_-integrin antibody, as indicated. VSVG, R-Ras and β_1_-integrin all colocalize within ruffles (yellow). Scale bar = 20 μm. Click here for file
