# Supplementary figures and images for: R-Ras regulates β1-integrin trafficking via effects on membrane ruffling and endocytosis
Source: BMC Cell Biol. 2010 Feb 18;11:14. doi: 10.1186/1471-2121-11-14 (PMC2830936; doi:10.1186/1471-2121-11-14)

GFP

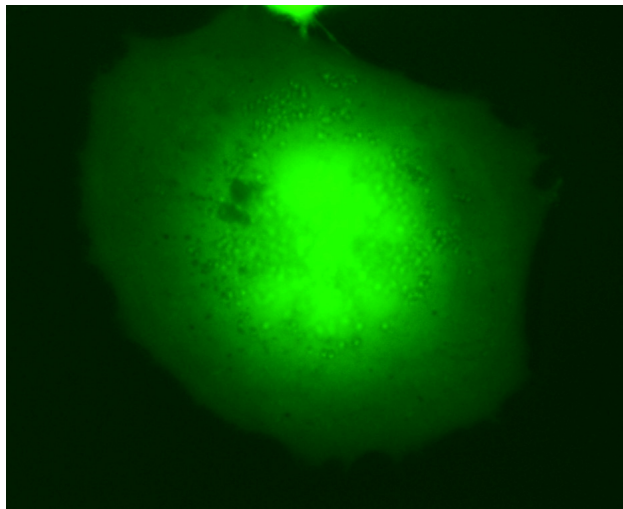

Supplement: Additional file 1 — GFP-alone control. Representative cell that was transfected with GFP alone showed no localization of the fluorophore to membranes. [file 1471-2121-11-14-S1.PDF]

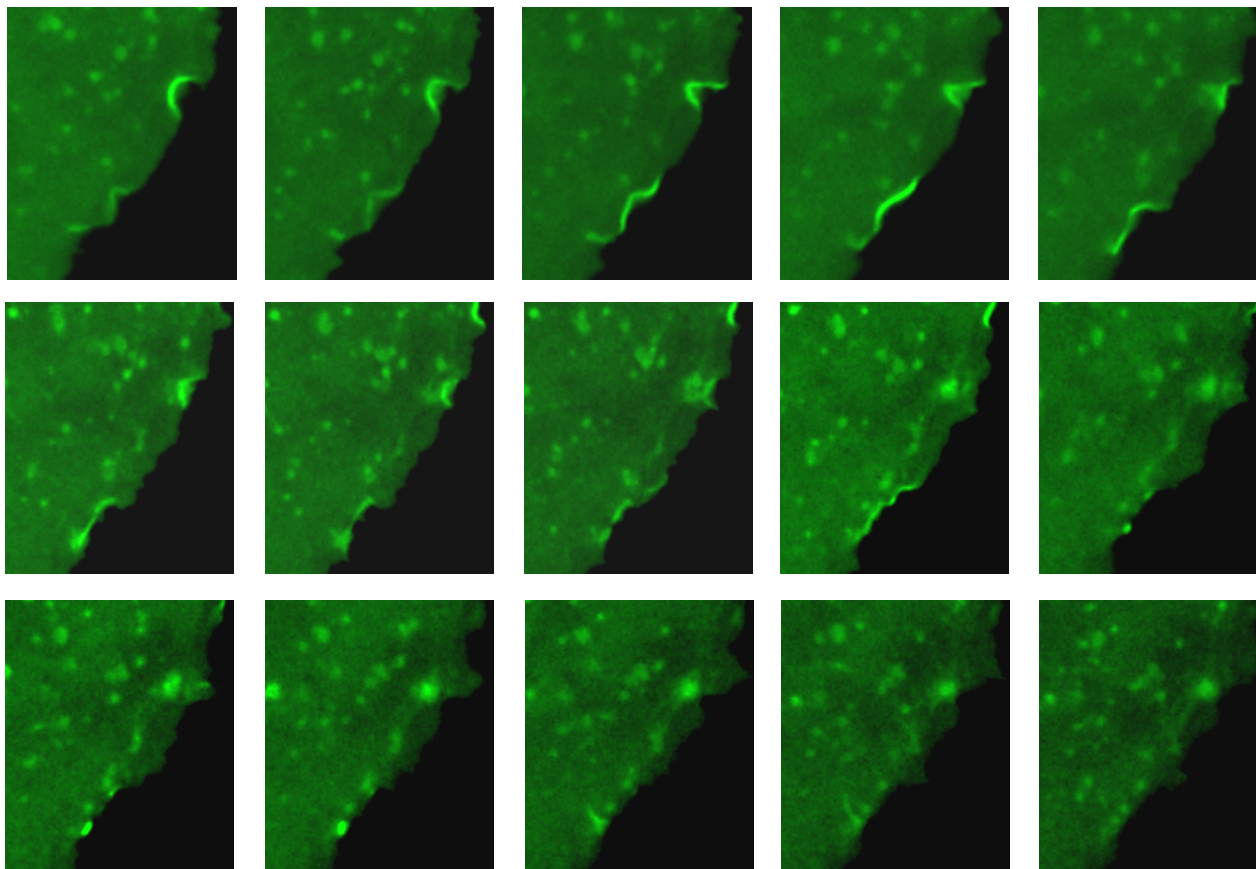

Supplement: Additional file 2 — Movie of GFP-R-Ras(wt) dynamics. Timelapse images of GFP-R-Ras(wt) localization. Images were acquired at 2 min intervals for 20 mins and played at 7 frames/sec. [file 1471-2121-11-14-S2.PDF]

GFP-R-Ras Wt

GFP-R-Ras 38V

GFP-R-Ras 41A

Lysotracker

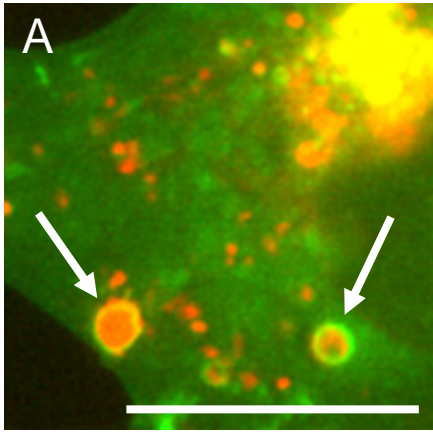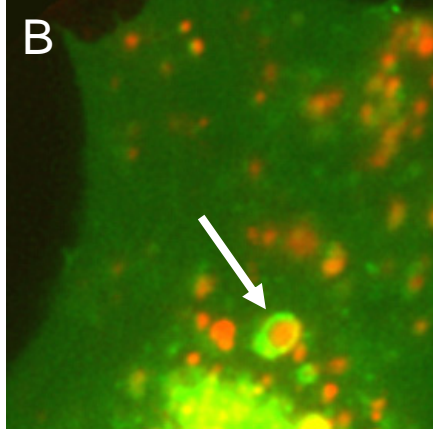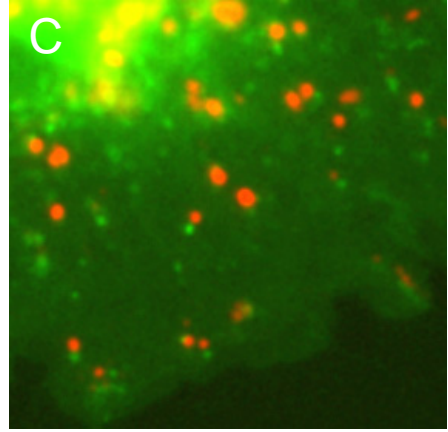

pCaveolin-1 (Y14)

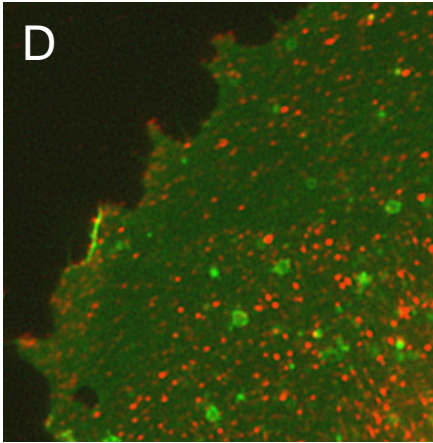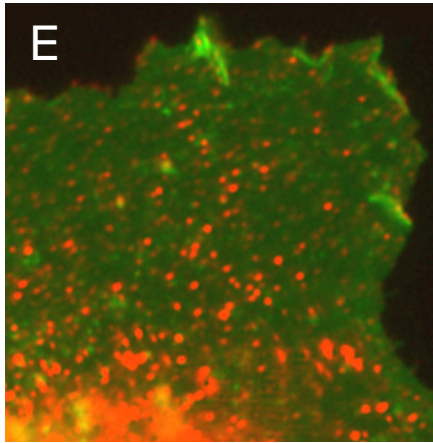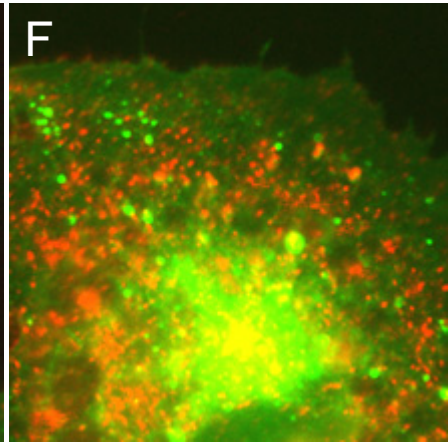

Supplement: Additional file 12 — Endocytosis of β1-integrin through the breakdown of ruffles. Inset view of movie 7 showing GFP-β1-integrin fluorescence breakdown into vesicles following ruffling. Images were acquired 30 seconds apart for 30 mins. Data was deconvolved using the no-neighbors algorithm. [file 1471-2121-11-14-S12.PDF]

GFP-VSVG  
Anti R-Ras

GFP- $\beta_1$ -integrin  
Anti R-Ras

GFP-VSVG  
Anti  $\beta_1$ -integrin

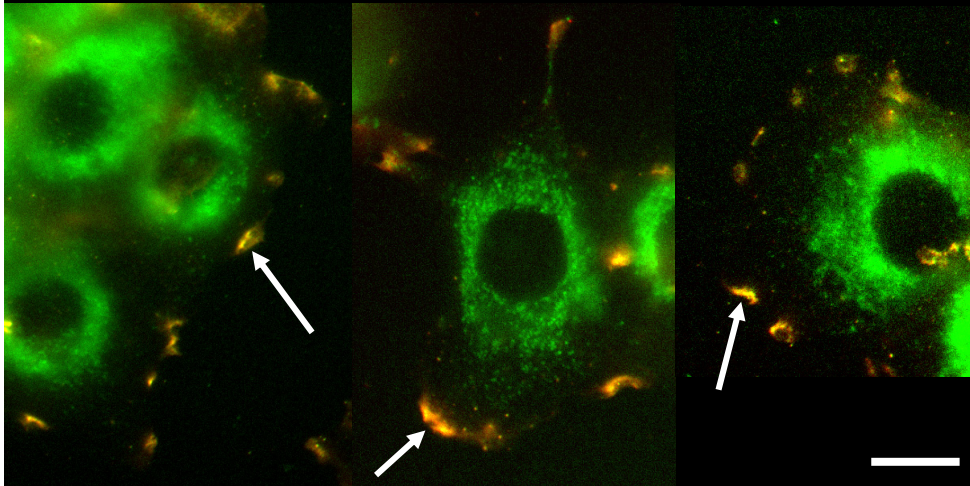

Supplement: Additional file 13 — GFP-VSVG. Movie of a GFP-VSVG transfected cell with a ruffling membrane. Images were acquired 30 seconds apart for 30 mins. Data was deconvolved using the no-neighbors algorithm. [file 1471-2121-11-14-S13.PDF]
